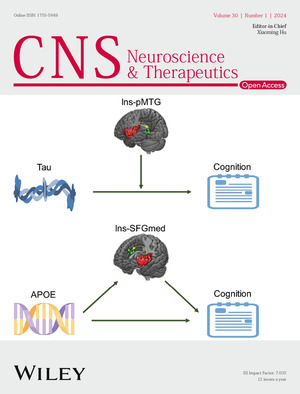# Additional Cover

**DOI:** 10.1111/cns.14624

**Published:** 2024-01-28

## Abstract

The cover image is based on the Original Article *The relationship between APOE genotype, CSF Tau and cognition across the Alzheimer’s disease spectrum, moderation and mediation role of insula network connectivity* by Yao Zhu et al., https://doi.org/10.1111/cns.14401.